# Authors’ Response to Peer Reviews of “Seroprevalence of SARS-CoV-2 in Niger State: Pilot Cross-Sectional Study”

**DOI:** 10.2196/50515

**Published:** 2023-10-17

**Authors:** Hussaini Majiya, Mohammed Aliyu-Paiko, Vincent Tochukwu Balogu, Dickson Achimugu Musa, Ibrahim Maikudi Salihu, Abdullahi Abubakar Kawu, Ishaku Yakubu Bashir, Aishat Rabiu Sani, John Baba, Amina Tako Muhammad, Fatimah Ladidi Jibril, Ezekiel Bala, Nuhu George Obaje, Yahaya Badeggi Aliyu, Ramatu Gogo Muhammad, Hadiza Mohammed, Usman Naji Gimba, Abduljelili Uthman, Hadiza Muhammad Liman, Sule Alfa Alhaji, Joseph Kolo James, Muhammad Muhammad Makusidi, Mohammed Danasabe Isah, Ibrahim Abdullahi, Umar Ndagi, Bala Waziri, Chindo Ibrahim Bisallah, Naomi John Dadi-Mamud, Kolo Ibrahim, Abu Kasim Adamu

**Affiliations:** 1 Department of Microbiology, Ibrahim Badamasi Babangida University, Lapai, Nigeria; 2 Center for Applied Sciences and Technology Research, Ibrahim Badamasi Babangida University, Lapai, Nigeria; 3 Trans-Saharan Disease Research Center, Ibrahim Badamasi Babangida University, Lapai, Nigeria; 4 Department of Biochemistry, Ibrahim Badamasi Babangida University, Lapai, Nigeria; 5 Department of Biology, Ibrahim Badamasi Babangida University, Lapai, Nigeria; 6 Department of Computer Science, Ibrahim Badamasi Babangida University, Lapai, Nigeria; 7 Department of Geography, Ibrahim Badamasi Babangida University, Lapai, Nigeria; 8 Department of Mathematics, Ibrahim Badamasi Babangida University, Lapai, Nigeria; 9 General Hospital, Minna, Nigeria; 10 Niger State Ministry of Health, Minna, Nigeria; 11 Ibrahim Badamasi Babangida Specialised Hospital, Minna, Nigeria

**Keywords:** COVID-19, pandemic, SARS-CoV-2, seroprevalence, serology, epidemiology, Niger State, Nigeria, COVID-19 testing, social distancing

*This is the authors’ response to peer-review reports for “Seroprevalence of SARS-CoV-2 in Niger State: Pilot Cross-Sectional Study.”*

## Round 1 Review

Thank you for going through and reviewing our manuscript. We have corrected, changed, and included most of the things you suggested. Please find as follows the specific responses to each of the comments made by the reviewers. Responses are placed immediately after each comment made by the reviewers.

### Reviewer S [1]

This is a pilot study [2] to determine the COVID-19 seroprevalence, patterns, dynamics, and risk factors in Niger State, Nigeria. The study design is a cross-sectional survey using clustered, stratified random sampling over 5 days; the prevalence was measured by detecting antibodies.

Major point: the study design uses clustered, stratified random sampling. The authors haven’t described the clusters or stratification. However, I understand this as study participants were allowed to have different, but known, probabilities of being selected for the sample. This is different to study designs where participants are selected with equal probability. However, none of the analyses presented in the manuscript accounted for this different probability of selection; all the analyses have assumed an equal probability of selection. This is a fundamental mistake of the analysis. This invalidates all the results presented in the manuscript. The term “sampling weights” is not used at all.

Response: Details were added to the sampling strategies in the Methods and Results sections as requested.

Other considerations:

- The justification for this pilot study is unclear. Specifically, what will be the full study that corresponds to this pilot? Since the COVID-19 situation changes rapidly, can the lessons from this study be used for designing a full study at a later stage?
- Response: This is a pilot study and was aimed at giving a quick feel of the COVID-19 situation at the time before the follow-up study, which can give the status and pattern of COVID-19 in the state.

- Some of the people sampled have not consented. How do they fill those gaps? Did they sample someone else in those places? What was the response rate as a measure of sampling bias in estimating prevalence?

- Response: All people that participated consented. More details were added to the sampling strategies in the Methods to give more clarifications.

- The inclusion and exclusion criteria are not given. The presented results are simple percentages from participants.

- Response: The age range covered all people and other stratifications, and therefore, unless they did not consent to the participation, all residents could be approached to participate and sampled. The exclusion criterion was not consenting to participate; the inclusion criterion was consenting to participate.

- The stratification is by place of residence (2 groups), gender (2 groups), occupation (unknown number of groups), and age (unknown number of groups). Therefore the number of strata should be large, although unknown to me. I wonder what could be the justification of these strata that must have resulted in a very small number of people per strata given the total sample size of 185.

- Response: More details were added to the sampling strategies in the Methods to give more clarifications.

- There are multiple places that require references (eg, second paragraph under section 2.4).

- Response: References were added as requested.

- Not sure what the value is of lots of bar graphs. Almost all of the information in those graphs is already in the text.

- Response: The Results are now summarized in tables in the revised manuscript. The results in the bar graphs were removed.

- The text needs revising in some places. For example, the first 1.5 paragraphs under section 3.2 do not belong in the Results section. Two of the subfigures in Figure 3 have been cited but mixed up in the second paragraph of that section.

- Response: The manuscript was revised as suggested and errors were corrected.

- Have they considered the incubation period needed to develop antibodies when interpreting the calculated percentages as prevalence?

- Response: Yes, we considered the incubation period needed to develop antibodies when doing the calculations; this is clear in the revised manuscript.

- Authors have determined the sensitivity and specificity as 100% for test kits; this was using the results from 15 individuals. I am skeptical to accept that in the absence of CIs.

- Response: The sample size of the participants and the small number of kits for validation were some of the limitations of the study; these were stated in the revised manuscript.

### Reviewer AV [3]

#### General Comments

This article is a pilot study that was conducted to determine the prevalence, patterns, and dynamics of COVID-19 and the risk factors for contracting the disease in Niger State from June 26 to 30, 2020.

This study is a cross-sectional study and uses a clustered, stratified random sampling method. Only 185 participants were included in the study. The sample size is small.

Response: The sample size of the participants and the small number of kits for validation were some of the limitations of the study; these were stated in the revised manuscript.

The seroprevalence of COVID-19 was found to be 25.4% and 2.16% for the positive IgG and IgM, respectively. These seroprevalence results mean that herd immunity to COVID-19 has yet to be achieved, and the population is still susceptible to more infection and transmission of the virus.

#### Specific Comments

##### Major Comments

1. Samples were taken randomly from 185 participants for COVID-19 IgG and IgM rapid tests and questionnaires. Information on the number of patients included in the different sampling points is missing. Have serology results been confirmed by other techniques?

Response: More information was added in the Methods section to clarify more. The serology results were not confirmed by other methods, but the kits were validated by polymerase chain reaction.

2. The results are expressed as a percentage; it would be interesting to have the data on the number of samples or the number of patients.How many participants tested positive for only IgG and for both IgG and IgM?

Response: Absolute numbers were provided for the relative results (percentages) in the tables.

3. Bibliographic references are not formatted in the correct format.

Response: References were done as requested.

##### Minor Comments

4. Page 1: explain “NCDC”

Response: It is now explained in the revised manuscript. It means Nigeria Center for Disease Control.

5. Page 5: italicize *Chlamydia pneumoniae*, *Mycoplasma pneumoniae*, *Treponema pallidum*

Response: Noted, but that part was removed from the manuscript.

6. Page 9: add percent majority (61.62%)

Response: Done as requested

7. Page 11: explain “ATM”

Response: automated teller machine

8. Page 14: replace igM with IgG, “while the Kit detecting only IgM means that...”

Response: Done as requested

9. Page 19: explain “PPE”

Response: personal protective equipment

### Anonymous [4]

#### General Comments

This paper, “Seroprevalence of COVID-19 in Niger State: A Pilot Cross-Sectional Study” by Majiya et al, is valuable and worthy of publication. The paper describes the seroprevalence of COVID-19 in Niger State. The COVID-19 asymptomatic rate in the state was 46.81%. The study also observed that the chances of infection are almost the same for both urban and rural dwellers. Of great interest is the finding that health care workers and those who had contact with persons who traveled out of Nigeria in the last 6 months are twice as likely to be at risk of being infected with the virus. The paper is relevant and contributes to the knowledge of the epidemiology of the virus. However, one primary concern is that the information about the virus from which inferences were made in this paper seems outdated. There is a need for an update. Also, the work appears to be underpowered in terms of sample size.

Response: The manuscript was revised and updated with regard to the recent COVID-19 situation in Nigeria. The sample size of the participants and the small number of kits for validation were some of the limitations of the study; these were stated in the revised manuscript.

#### Specific Comments

1. The abstract is unusually extended; consider summarizing it, especially the results aspect.

Response: The abstract was summarized and shortened as suggested.

2. There is a need for editing and restructuring some sentences.

Response: The manuscript was revised and grammar checked.

3. Some long paragraphs have the same references; consider using other references as well.

Response: More references were added as suggested.

4. Give a reference or definition for your sampling technique and probably describe how you achieved your sample size.

Response: More sampling information including the sample size calculation was added in the Methods as requested.

5. Avoid repeating the methodology in the Discussion session.

Response: Repetitions were removed as requested.

6. Add references to back up your inferences.

Response: More references were added where necessary for the key findings and interpretations.

7. The authors should make inferences in light of observation and the literature; asymptomatic cases seem to foster community transmission. More so, isolation, quarantine, and lockdown, if need be, are some public health measures to halt transmission. I would instead advise that the authors make recommendations based on the data generated from the study.

Response: More references were added where necessary for the key findings and interpretations. Those earlier interpretations that contradict public health measures were all removed in the revised manuscript.
